# miRNA-1246, HOTAIR, and IL-39 signature as potential diagnostic biomarkers in breast cancer

**DOI:** 10.1016/j.ncrna.2023.02.002

**Published:** 2023-02-10

**Authors:** Amal K. Khaliefa, Ekram M. Desouky, Walaa G. Hozayen, Saeed M. Shaaban, Nabil A. Hasona

**Affiliations:** a Department of Biochemistry, Faculty of Science, Beni-Suef University, Salah Salim St., 62511, Beni-Suef, Egypt; b Oncology Department, Faculty of Medicine, Beni-Suef University, 62511, Beni-Suef, Egypt; c Beni Suef National University, Faculty of Science, Biochemistry Department, Beni Suef, 62511, Egypt

**Keywords:** miR-1246, HOTAIR, IL-39, Biomarker, Breast cancer

## Abstract

The molecular alterations in noncoding RNA can lead to a cellular storm that is correlated to higher mortality and morbidity rates and contributes to the progression and metastasis of cancer. Herein, we aim to evaluate the expression levels and correlations of microRNA-1246 (miR-1246), HOX transcript antisense RNA (HOTAIR), and interleukin-39 (IL-39) in patients with breast cancer (BC). In this study, 130 participants were recruited, including 90 breast cancer patients and 40 healthy control participants. Serum levels of miR-1246 and HOTAIR expression were assessed by quantitative real-time polymerase chain reaction (qRT-PCR). Also, the level of IL-39 expression was evaluated using a Western blot. All BC participants demonstrated a remarkable elevation in miR-1246 and HOTAIR expression levels. Moreover, IL-39 expression levels demonstrated a noticeable decline in BC patients. Furthermore, the differential expression fold of miR-1246 and HOTAIR revealed a strong positive correlation among breast cancer patients. In addition, a negative relationship between the IL-39 and the miR-1246 and HOTAIR differential expression was also noticed. This study revealed that HOTAIR/miR-1246 exerts an oncogenic impact in patients with breast cancer. The expression levels of circulation miR-1246, HOTAIR, and IL-39 could be considered early diagnostic biomarkers in BC patients.

## 1 Introduction

The most frequent cancer in women and the cause of death is breast cancer (BC) [1]. The prevalence of BC has increased over the past decade in many nations [2]. This rise is accelerating in Asia, Africa, and South America, where the disease has a shorter history. The majority of the cases of breast cancer in Egyptian women occur at advanced stages, making up about 37.6% of all malignant tumors [3]. The disparity in disease progression rates could be due to socioeconomic changes and the industrialization of societies [4]. Age, ethnicity, family history, increased alcohol consumption, extended duration of the first pregnancy, and genetic predisposition are the main risk factors for BC [5,6]. Moreover, Due to the tumor's variable clinical presentation and the prevalence of practically imperceptible micro-metastases, it is challenging to accurately diagnose BC patients [7]. Therefore, a sensitive and precise biomarker that can identify breast cancer in its early stages and determine prognosis is crucial.

Micro RNAs (miRNAs) are single-stranded RNA molecules with 22 nucleotides that regulate the expression of eukaryotic genes by targeting untranslated region (UTR) of mRNA, coding sequence, and gene promoters. Recent studies have revealed the critical roles of miRNAs in cell proliferation, differentiation, cell cycle, and migration [8]. Various malignancies, including BC, have been linked to the dysregulation of miRNAs [9,10]. MiR-1246 and HOTAIR expression profiles have been implicated in various diseases. In addition, miR-1246 and HOTAIR expression, which bind to target genes, play critical roles in metabolic reprogramming in BC implying that miR-1246 and HOTAIR distinguished promise for improving BC detection and have varying relationships with BC patients in their expression patterns.

A recently found microRNA known as miR-1246 (miR-1246) has been linked to several cancers, including breast cancer. The miR-1246 gene is found on chromosome II (2q31.1) of the human genome and functions as a transcriptional target of p53. The control of the cell cycle, apoptosis, and senescence are all affected by its expression [11]. In addition, the miR-1246 signaling was evidenced to influence the proliferation, motility, invasion, and metastasis of cancer cells [12,13]. Several malignancies, such as colon, lung, liver, and stomach, have miR-1246 identified as a proto-oncogene [11].

Regulatory RNAs, known as long noncoding RNAs (lncRNAs), can alter the stability, splicing, and translation of mRNA as well as the conformation of chromatin to either promote or inhibit gene expression. Additionally, recent studies have revealed the role of lncRNAs in metabolic regulation that can drive alterations in genetic programming in the proliferation and differentiation of the cell [[14], [15], [16], [17]].

On chromosome 12q13.13, in the region between the HOXC11 and HOXC12 genes, is a 2158-bp long lncRNA called HOX transcript antisense RNA (HOTAIR) [18]. The interaction of HOTAIR with the primary molecular pathways involved in BC carcinogenesis has been linked to the progression of BC [19,20]. Additionally, primary BC with a high risk of metastasis and a poor prognosis was shown to have aberrant HOTAIR expression. HOTAIR could be a significant predictor of BC tumor development, according to Ref. [21].

Imbalances between pre- and anti-inflammatory cytokines significantly influence the onset and progression of cancer. Heterodimeric cytokine, IL-39 was firstly discovered by Wang et al. [22], as a proinflammatory cytokine and significantly increased and mediated inflammatory responses in lupus mice. IL-39 study in humans currently appears to have conflicting outcomes. On the other hand, Weng et al. [23], recently reported that circulating levels of IL-39 significantly decreased in autoimmune thyroid diseases. However, the potential signature of IL-39 in breast cancer is still unknown.

MiR-1246 and HOTAIR have emerged as significant regulators among the numerous miRNAs regulating inflammatory adipokines and diseases. Thus, shedding light on miR-1246 and HOTAIR's relationship with IL-39 as novel inflammatory mediators for detecting breast cancer growth and metastasis is crucial. This study aimed to assess the expression levels of miR-1246, HOTAIR, and IL-39 in blood samples of BC patients as a potential early diagnostic biomarker and scrutinized their differential expression with PR/ER status.

## 2 Subjects and methods

### 2.1 Subjects

With an average age of 45.71 ± 10.05 years (mean ± SD), 130 female participants were enrolled in this study from the oncology Department, Faculty of Medicine, Beni-Suef University. The participants were split into four groups: Group 1 BCE patients with stage I (n = 30), Group 2 BCE patients with stage II (n = 30), Group 3 BCE patients with stage III (n = 30), and Group 4 healthy control individuals (n = 40). None of these healthy volunteers had ever had a diagnosis of cancer, high blood pressure, diabetes, or any other illness. All BC diagnosed patients confirmed pathologically after a positive mammogram, tumor characteristics were noted: tumor size, kind, grade, tumor-node-metastasis (TNM) stage, and progesterone/Estrogen receptor status. Patients undergoing chemotherapy or radiation treatment or who have an acute infection are eligible for exclusion from participation in the current study. Written informed consent was signed by all study participants prior to taking part in the study. Additionally, the study protocol met the requirements of the Helsinki Declaration and was approved by the Faculty of Medicine's Ethics Committee at Beni-Suef University (FMBSUREC/02102022/Hasona).

### 2.2 Blood sample handling

Participants' blood samples were taken in plain tubes (5 mL each). To remove cell debris, two centrifugation processes were performed in quick succession (15,000 g for 10 min at 4 °C and 20,000 g for 3 min at 4 °C). Serum samples were stored at −80 °C until RNA extraction and IL-39 Western blot analysis.

### 2.3 Serum HOTAIR, and miR-1246 assay

A miRNeasy extraction kit was used to extract the RNA from a total sample volume of 100 μL serum (Qiagen, Valencia, CA, USA). Before reverse transcription of the RNA into cDNA using the DNase Max Kit, the sample was treated with DNase to eliminate any sources of DNA after extraction. Amplification of the cDNA was carried out by SYBR Green and the following primer assay numbers were: miR-1246 (462,575), HOTAIR (LPH07360A), RNU6B (001093), and GAPDH (LPH31725A). The differential expression was assessed by the 2^−ΔΔCt^ method [24].

### 2.4 IL-39 expression assay

A Bradford assay was performed to determine protein concentration. 20 μg of proteins underwent SDS-PAGE gel electrophoresis before being transferred to nitrocellulose membranes. The membranes were blocked, then incubated with primary antibodies for IL-39 (cat. no. 9990-IL; R&D systems bio-techne) overnight at 4ᴼC. The membranes were incubated with secondary antibodies, developed with an enhanced chemiluminescence kit provided by Bio-Rad Inc (Catalog #163–2086), and the bands were quantified utilizing ImageJ.

### 2.5 Statistical analysis

The SPSS 22 was used for the statistical analysis of data (SPSS, Inc., Chicago, IL, USA). All statistical data are displayed as means ± standard error means (SEM). The post-hoc multiple comparisons tests (Duncan) were employed for pairwise comparisons. The Pearson correlation coefficient was used to evaluate the relationships between the variables under study. Receiver Operating Characteristic (ROC) curves were employed to assess the HOTAIR, miR-1246 and IL-39 diagnostic performances.

## 3 Results

In this study, 130 participants were recruited, including 90 breast cancer patients (30 Stage I, 30 Stage II, and 30 Stage III) and 40 healthy control participants (Table 1).

**Table 1:** Demographic characteristics of the studied groups.

Variables	Healthy participants (n = 40)	All breast cancer Patients (n = 90)	Stage	Stage II (n = 30)	Stage III (n = 30)
			I (n = 30)		
Age (mean ± SD, year)	37.48 ± 7.78		41.86 ± 7.02	48.83 ± 6.86	55.79 ± 7.57

Menstrual history:	39	60	23	12	16
Premenopausal	1	30	7	18	14
Postmenopausal					
PR/ER status:		11 (12.2%)	2(6.7%)	4(13.3%)	5(16.7)
Positive		79 (87.8%)	28(93.3%)	26(86.7)	25(83.3%)
Negative					
Tumor type:		82(91.11%)	26(86.67%)	28(93.33%)	28(93.33%)
Duct		8(8.89%)	4(13.33%)	2(6.67)	2(6.67%)
Lobular					
Metastasis:		90	30	30	30
M0					

Estrogen receptor (ER); Progesterone receptor (PR).

Table 2 shows the levels of miR-1246 and HOTAIR expression in the serum of all recruited participants. Compared to matched healthy females, breast cancer patients had a higher mean in relative levels of serum miR-1246 and HOTAIR (Table 2). miR-1246 and HOTAIR expression exhibited a remarkable elevation among all stages of BC patients. As demonstrated in Table 2, both miR-1246 and HOTAIR expression were significantly higher in stage III than in stages I and II (Table 2).

**Table 2:** Expression levels of miR-1246and HOTAIR among healthy and breast stage study participants.

Variables	miR-1246	HOTAIR
Healthy control	0.92 ± 0.02^a^	0.91 ± 0.02^a^
Stage I	1.52 ± 0.10^b^	1.69 ± 0.12^b^
Stage II	2.43 ± 0.15^c^	2.43 ± 0.19^c^
Stage III	3.40 ± 0.18^d^	3.86 ± 0.21^d^
F-ratio	71.61	65.07
P- value	<0.001	<0.001

Data were expressed as mean ± SEM; According to the Duncan multiple range test, the different letters indicate statistical significance different means. miR-1246; microRNA-1246. HOTAIR; HOX transcript antisense RNA. IL-39; Interleukin-39.

**Fig. 1:**
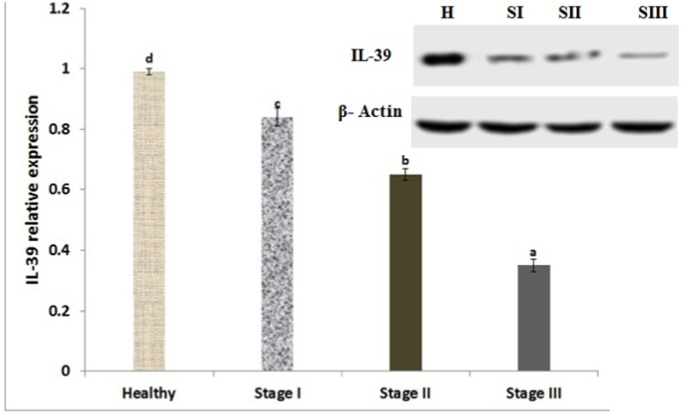
IL-39 expression levels among study participants. The different letters indicate statistical significance different means.

**Fig. 2:**
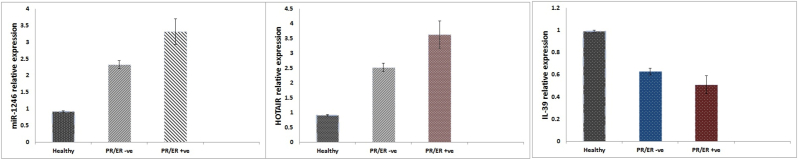
(a, b, c) Expression levels of miR-1246, HOTAIR, and IL-39 among PR/ER status study participants.

Serum IL-39 expression level showed a noticeably (P < 0.001) down-regulation in the BC patients versus the healthy participants. IL-39 demonstrated a noticeable decrease in its differential expression along with BC stages (Fig. 1). According to Fig. 1, stage III patients had the lowest expression patterns, which were noticeably different from those in stages I and II. Additionally, miR-1246 and HOTAIR demonstrated a noticeable elevation in its differential expression in PR/ER positive participants (Fig. 2a, 2b). While, IL-39 showed a noticeable decline in its expression in PR/ER positive participants as compared to PR/ER negative (Fig. 2 c).

The differential expression fold of miR-1246 and HOTAIR revealed a strong positive correlation among breast cancer patients (Table 3). In addition, a negative relationship between the IL-39 and the miR-1246 and HOTAIR differential expression was also noticed (Table 3).

**Table 3:** Pearson correlation coefficient between miR-1246, HOTAIR, and IL-39.

Parameter	miR-1246		HOTAIR		IL-39	
	R	*P*	R	*P*	R	*P*
miR-1246	–	–	0.856^a^	<0.001	−0.773^a^	<0.001
HOTAIR	0.856^a^	<0.001	–	–	−0.769^a^	<0.001
IL-39	−0.773^a^	<0.001	−0.769^a^	<0.001	–	–

a Significant linear correlation at P < 0.01 (2-tailed). miR-1246; microRNA-1246. HOTAIR; HOX transcript antisense RNA. IL-39; Interleukin-39.

**Fig. 3:**
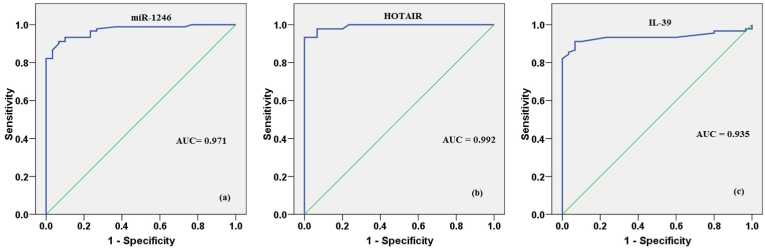
(a, b, c) ROC analysis regarding miR-1246, HOTAIR and IL-39 between breast cancer patients and healthy participants.

With an AUC of 0.971, a 95% confidence interval of 0.946–0.997, a sensitivity of 91.11%, and 93.33% specificity, serum miR-1246 distinguished breast cancer patients from healthy volunteers (Fig. 3a). Likewise, the AUC concerning the serum HOTAIR was 0.992, with P < 0.001, a sensitivity of 93.33%, and 100% specificity (Fig. 3b). Moreover, the ROC for serum IL-39 expression showed AUC = 0.935, 95% confidence interval 0.888–0.981, with 91.11% sensitivity and 93.33% specificity (Fig. 3c).

## 4 Discussion

Inadequate management and control of breast cancer predispose patients to many complications. Thus, this opens new windows for exploring and scrutinizing novel candidate biomarkers more effective in diagnosis. Herein, we evaluated the expression level of miR-1246 and HOTAIR in blood samples of BC patients as a potential early diagnostic biomarker and scrutinized their differential expression with PR/ER status.

Numerous types of miRNAs-derived exosomes have been reported in different types of malignancies, particularly stomach [25], liver [26], and lung [27], since identified in patients' serum with lymphoma [28], which makes them good biomarkers for future clinical purposes. Yet, only a small number of ncRNAs have been identified as possible biomarkers in human serum, and unexpectedly, little is known about their relationships with inflammatory biomarkers in BC.

Herein, the miR-1246/HOTAIR differential expression levels were assessed in female patients with BC and compared with that in healthy participants. All participants with BC showed up-regulation with significant differences in miR-1246 and HOTAIR5 expression. In addition, miR-1246 and HOTAIR expression exhibited a remarkable elevation among all stages of BC patients, both miR-1246 and HOTAIR expression were significantly higher in stage III than in stages I and II. Additionally, The differential expression fold of miR-1246 and HOTAIR revealed a strong positive correlation among breast cancer patients.

Here, receiver operating characteristic (ROC) curves were employed to assess the HOTAIR and miR-1246 diagnostic performances. Distinguishing participants with BC from healthy participants, the AUC was 0.992 and 0.999, respectively (P < 0.001), which implies the impact of the differential expression of miR-1246 and HOTAIR as suitable diagnostic biomarkers for predicting the progress of breast cancer.

Consistence with the results of the current study, Bott et al. [29] and Jang et al. [30] reported that BC exhibit a remarkable high expression level of miR-1246. Furthermore, studies employing cancer tissues and cell lines have thus far described the role of miR-1246 in cancer. Li et al. [31] and Zhong et al. [32] concluded that elevated levels of miR-1246 expression are a characteristic of metastatic breast cancer. Yu et al. [33] concluded that the elevated levels of miR-1246 expression may aid melanoma cell viability and metastasis. miR-1246 contributes to curcumin's reduced radiation sensitivity and anticancer properties. It has been reported that miR-1246 targets p53 directly and prevents p53 translation [34]. Furthermore, Du et al. [35] demonstrated that elevated levels of miR-1246 expression block thrombospondin-2 in uterine cervical carcinoma, which controls cell adhesion and migration through the hydrolysis of the extracellular matrix and a signaling cascade. The previous investigations strengthen our notion about the oncogenic role of miR-1246 in breast cancer and its influence on the tumor progression in the adjacent and distant normal cells.

Regarding overexpression of HOTAIR in patients with breast cancer, agreed with Zhang et al. [36]; Xue et al. [37], and Arshi et al. [38], who stated that the HOTAIR expression was significantly higher in patients with breast cancer and associated with its progression. In addition, Srensen et al. [39] revealed a conclusive relationship between HOTAIR and the risk of metastasis in breast cancer and consider HOTAIR as a standalone biomarker in the diagnosis of breast cancer. The HOTAIR tends to promote the progression of carcinogenesis by combination with polycomb repressive complex 2 (PRC2), which initiates the methylation of histone H3K27 that is involved in dampening the transcription of Wnt inhibitory factor 1 (WIF-1), thereby activating Wnt/β-catenin pathway in BC. Similarly, Li et al. [40] revealed that HOTAIR increases c-Myc via the Wnt/-catenin signaling pathway, which is significant for leukemia cell proliferation, invasion, and metastasis. Recently, a significant association between HOTAIR overexpression and the alteration of microRNA expression was reported [41,42]. Liu et al. [43], Zhao et al. [44], and Zhou et al. [45] reported that The crucible roles of HOTAIR expression in miR-143–3p/BCL2 and miR-20a-5p/HMGA2 axes, thereby controlling the genes involved in metastasis and progression of cancer.

Interleukins have been assigned a profound role in the homeostasis of normal cells and participating in the carcinogenesis pathway. A recently discovered cytokine called IL-39 has not yet investigated for its function in the pathophysiology of neoplasia. According to the results of our investigation, the levels of IL-39 were considerably lower in BC patients than in healthy controls. Furthermore, circulation IL-39 in breast cancer patients was found to correlate negatively with their miR-1246 and HOTAIR levels. IL-39 may play a crucible role in many disorders, according to several investigations, which has sparked a lot of debate among scientists.

Recent studies have demonstrated lowered IL-39 expression in autoimmune thyroid diseases [23]. Meanwhile, IL-39 impedes the progression and survival of cancer by promoting the apoptosis of T24 bladder cancer cells and pancreatic cancer [46,47]. Nevertheless, Luo et al. [48] reported higher IL-39 expression in acute coronary syndrome.

Although the correlation between stages and metastasis of cancer and intracellular ncRNA has been limited so far, the different investigations provided conflicting results, with a significant proportion of ncRNAs and inflammatory markers upregulated in one study but down-regulated in another. Therefore, characterization of the complex relationship between ncRNAs, inflammatory markers, and target genes was needed to aid in enhancing breast cancer treatment and early detection. However, this study has some limitations, including the sample size and the impacts of these markers on putative target genes and pathways of carcinogenesis.

## 5 Conclusion

In conclusion, this study revealed a positive correlation between HOTAIR and miR-1246 expression and exerts an oncogenic impact in patients with breast cancer, and reported a decline in IL-39 levels in patients with breast cancer. Serum IL-39 provided potential early diagnostic performance indicators of breast cancer that warrant further evaluation.

## Funding

The authors declares that they had received no funding for the research reported.

## Ethics approval and consent to participate

This study was conducted in compliance with the Declaration of Helsinki, and approved by the Faculty of Medicine's Ethics Committee at Beni-Suef University.

## Availability of data and materials

All data generated or analysed during this study are included in this published article.

## CRediT authorship contribution statement

Amal K.Khaliefa, Nabil A.Hasona, and Saeed M.Shaaban contributed to the study's conception and design. Material preparation, data collection and analysis were performed by Nabil A.Hasona, Walaa G.Hozayen and Ekram M.Desouky The first draft of the manuscript was written by Nabil A.Hasona and Walaa G.Hozayen. All authors read and approved the final manuscript.

## Declaration of competing interest

The authors declare no conflicts of interest.
